# Family in Rehabilitation, Empowering Carers for Improved Malnutrition Outcomes: Protocol for the FREER Pilot Study

**DOI:** 10.2196/12647

**Published:** 2019-04-30

**Authors:** Skye Marshall, Barbara S van der Meij, Rachel Milte, Clare E Collins, Marian AE de van der Schueren, Mark Banbury, Molly M Warner, Elizabeth Isenring

**Affiliations:** 1 Bond University Nutrition & Dietetics Research Group Faculty of Health Sciences and Medicine Bond University Robina Australia; 2 Dietetics and Foodservices Mater Health Brisbane Australia; 3 Mater Research Institute University of Queensland Brisbane Australia; 4 Institute for Choice University of South Australia Adelaide Australia; 5 Faculty of Health and Medicine University of Newcastle Callaghan Australia; 6 Priority Research Centre in Physical Activity and Nutrition The University of Newcastle Callaghan Australia; 7 Department of Nutrition and Dietetics Amsterdam UMC Vrije Universiteit Amsterdam Netherlands; 8 Department of Nutrition and Dietetics HAN University of Applied Sciences Amsterdam Netherlands; 9 Northern NSW Local Health District NSW Health Tweed Heads Australia; 10 Faculty of Health Sciences and Medicine Bond University Robina Australia

**Keywords:** carers, protein-energy malnutrition, telehealth, intervention, pilot study, older adults, subacute, rehabilitation, aged

## Abstract

**Background:**

Interventions to improve the nutritional status of older adults and the integration of formal and family care systems are critical research areas to improve the independence and health of aging communities and are particularly relevant in the rehabilitation setting.

**Objective:**

The primary outcome aimed to determine if the FREER (Family in Rehabilitation: EmpowERing Carers for improved malnutrition outcomes) intervention in malnourished older adults during and postrehabilitation improve nutritional status, physical function, quality of life, service satisfaction, and hospital and aged care admission rates up to 3 months postdischarge, compared with usual care. Secondary outcomes evaluated include family carer burden, carer services satisfaction, and patient and carer experiences. This pilot study will also assess feasibility and intervention fidelity to inform a larger randomized controlled trial.

**Methods:**

This protocol is for a mixed-methods two-arm historically-controlled prospective pilot study intervention. The historical control group has 30 participants, and the pilot intervention group aims to recruit 30 patient-carer pairs. The FREER intervention delivers nutrition counseling during rehabilitation, 3 months of postdischarge telehealth follow-up, and provides supportive resources using a novel model of patient-centered and carer-centered nutrition care. The primary outcome is nutritional status measured by the Scored Patient-Generated Subjective Global Assessment Score. Qualitative outcomes such as experiences and perceptions of value will be measured using semistructured interviews followed by thematic analysis. The process evaluation addresses intervention fidelity and feasibility.

**Results:**

Recruitment commenced on July 4, 2018, and is ongoing with eight patient-carer pairs recruited at the time of manuscript submission.

**Conclusions:**

This research will inform a larger randomized controlled trial, with potential for translation to health service policies and new models of dietetic care to support the optimization of nutritional status across a continuum of nutrition care from rehabilitation to home.

**Trial Registration:**

Australian New Zealand Clinical Trials Registry Number (ACTRN) 12618000338268; https://www.anzctr.org.au/Trial/Registration/TrialReview.aspx?id=374608&isReview=true (Archived by WebCite at http://www.webcitation.org/74gtZplU2).

**International Registered Report Identifier (IRRID):**

DERR1-10.2196/12647

## Introduction

### Background

In older Australians, protein-energy malnutrition (PEM) is highly prevalent and a strong independent contributor to poor health, but is preventable and treatable [1-4]. PEM is defined as the unintentional and preventable loss of lean tissues such as muscle, blood and immune cells, and viscera, with or without fat loss, due to prolonged inadequate dietary intake or uptake of protein and energy [1]. Although PEM may occur at any age, it is most prevalent in older adults due to the higher prevalence of PEM risk factors such as multimorbidity and polypharmacy, and the physiological and social changes that occur during the aging process [5]. A sufficient increase in protein and energy intake and uptake to meet individualized requirements will cease the loss of lean tissues and reverse PEM, except in severe cachectic states [1]. However, encouraging malnourished older adults to consume appropriate types and quantities of foods encounters many diverse barriers due to its deeply complex physiological, socio-economic, and environmental risk factors, as well as unique presentation in each individual [5,6]. Individualized and long-term nutrition support is required to overcome these barriers and enable the older adult to improve their nutritional status [3,7]. Thus, the model of care adopted by many hospitals, which involves short-term treatment by health professionals during a health care admission only, is usually insufficient to effectively treat PEM [5,8].

Interventions to improve the nutritional status of older adults and the integration of formal and family care systems are critical research areas of the United Nations (items 2.6.10 and 2.10.7) [9], and implementing these approaches in rehabilitation facilities is of primary importance in Australia. Australian rehabilitation units have the highest prevalence of PEM internationally (45%-65% versus 30%-45% in the United States, Europe, and Asia when using the same diagnostic tool, N=17 studies, N=4591 participants) [10]. Although the goal of rehabilitation is to increase independence, observational research identified that older patients admitted to rehabilitation with PEM and receiving usual care were being discharged to the community with PEM, where they remained malnourished for at least 12 weeks in their own homes [10]. A recent meta-analysis found the prevalence of PEM in older Australians living in their own homes is 6% (95% CI, 4.4%-8.2%), which represents 228,000 malnourished older adults in 2017 [11]. Further downstream health consequences are severe, where PEM significantly predicts decreased physical function, institutionalization, and rehospitalization, poor quality of life, and death [3,10,12].

Family carers are an untapped resource and feasible group of people eager to support malnourished patients in the long-term [13]. There is a direct causal link between poor nutrition knowledge of family carers and increased PEM risk in older adults [14]. Conversely, studies have found that empowering family carers of malnourished older adults living at home (via training, education, and follow-up) can improve the nutritional status, quality of life, and physical function of the older adult, without increasing carer burden [15,16]. A qualitative study found that family carers believe it is the responsibility of rehabilitation staff to ensure the family carers are engaged as key members of the nutrition care team and that their preexisting caring relationship with the older adult is recognized and respected [13]. The qualitative study further identified the preferred method of engagement was via telephone, which is supported by a recent systematic review and meta-analysis, which found that telehealth was a feasible and effective method to provide PEM treatment post-hospital discharge [17]. The FREER (Family in Rehabilitation: EmpowERing Carers for improved malnutrition outcomes) pilot study will be the first to translate this evidence as a patient- and carer-centered model of care for the rehabilitation and post-rehabilitation older adult setting.

Therefore, this study aims to (1) determine if the FREER intervention in malnourished older adults during and postrehabilitation improves nutritional status, physical function, quality of life, service satisfaction, and hospital and aged care admission rates up to 3-months post discharge, compared with usual care, (2) evaluate secondary outcomes including carer burden, carer service satisfaction, and patient and carer experiences, and (3) assess the feasibility and intervention fidelity to inform a larger randomized controlled trial (RCT).

## Methods

### Study Design

This is the protocol for a pragmatic mixed-methods two-arm historically-controlled prospective pilot intervention study. This protocol has been reported according to the Standard Protocol Items: Recommendations for Intervention Trials (SPIRIT) 2013 Checklist [18] as well as the Template for Intervention Description and Replication (TIDieR) Checklist [19]. The FREER pilot study has been prospectively registered with the Australian New Zealand Clinical Trials Registry Number (ACTRN12618000338268).

### Participants and Setting

The recruitment site will be a single government-funded rehabilitation unit (34 beds, average length of stay of 22 days) in rural New South Wales, Australia (conveniently sampled), which is co-located with an acute care hospital. Patients are usually transferred from acute care to the rehabilitation units if they are not independent enough to return to the community after acute illness. However, admissions from the community are also accepted and are usually for the management of chronic conditions such as Parkinson disease or chronic obstructive pulmonary disease. The rehabilitation unit does not admit patients with preexisting dementia or severe cognitive impairment and provides services to general rehabilitation patients (ie, does not have age or diagnosis-specific admission criteria). Both eligible patients and family carers will be recruited according to the eligibility criteria (Textbox 1). Reflecting the pragmatic nature of the study, palliative patients and patients with unexpected discharge to residential aged care are included in the study. According to the Patient Generated Subjective Global Assessment (PG-SGA), patients rated as B (suspected of malnutrition or moderately malnourished) and C (severely malnourished) will both be considered as having PEM. Patients rated as A (well-nourished) will be excluded.

Potentially eligible patients identified by the rehabilitation clinical team using existing malnutrition screening upon admission will be placed on a high protein, high energy diet code, and referred to the study accredited practicing dietitian (MW, herein referred to as the “study dietitian” throughout) for full eligibility screening. Additionally, the study dietitian will attend team ward meetings and discuss patient lists with the rehabilitation clinical team. Ineligible or nonconsenting patients will receive usual care and will not be affiliated with the study. Consecutive rolling recruitment will continue over a maximum of 12 months. Informed by historical control group data [10], it is expected there will be approximately 90 eligible and consenting patients admitted to the rehabilitation unit per year (approximately 280 admissions per year, 40% of patients eligible, and 80% consent rate). Therefore, during the recruitment period, the minimum sample size of 30 patient-carer pairs will be met.

### Historical Control Group and Usual Care

Participants in a prospective observational study conducted 2013-2014 will act as a historical control group [10]. The historical control participants were recruited from 2 government-funded rural rehabilitation units in New South Wales (n=14 and n=16 participants respectively), one of which is the study site for the FREER pilot study, with participants having the same eligibility criteria as FREER. They had a mean age of 80 years and 57% were female [10]. The historical control group received usual care, which included being placed on a standard high protein-high energy diet [20] during admission and receiving standard nutrition support from the existing rehabilitation dietitian, but only if referred by the usual clinical pathways. The usual care provided to the historical control group has not changed at the time of the FREER pilot study and therefore is the same care provided to patients’ ineligible for FREER. Although the service was available, no participants in the historical control group received outpatient follow-up by a community dietitian [10]. Family carers of patients in the control group were not engaged specifically but may have been involved in some discussions with the rehabilitation dietitian during their care recipient’s admission. The outcomes in the historical control group have been published elsewhere [10,12].

**Inclusion and exclusion criteria.**

Inclusion

- Adults (≥65 years) admitted to rehabilitation with protein-energy malnutrition diagnosed by the Patient Generated Subjective Global Assessment
- Having a family carer (≥18 years). Family carers will be considered persons (including family or friends) who assist with activities of daily living up until the point of hospital admission, with no financial reimbursement for caring duties beyond a carers pension, with contact with the patient of ≥4 times per week, either in person or by telephone
- Family carer is English speaking and able to act as translator for the patient if the patient is non-English speaking
- Family carers do not have any health-related eligibility criteria applied; however, need to have sufficient independence to assist the patients with activities of daily living

Exclusion

- Patient and/or carer are unable to give consent
- Patient is receiving enteral or parenteral feeding
- Discharge is planned in <6 days from date of eligibility screening
- Patients living in residential aged care prior to rehabilitation admission are excluded. However, patients previously community-dwelling but discharged to residential aged care will be included using an intention-to-treat approach
- The patient and/or carer do not live in the local area. For example, admitted during holiday, or plan to move away from the local area (1.5 hours drive from the unit) within 3 months postdischarge

### Sample Size

As a pilot intervention study, the sample size was chosen to reflect resources and funding availability, as well as aiming to match control and intervention participants in a 1:1 ratio. Therefore, the current pilot intervention will aim to match the historical control group sample size (n=30), for a final sample of n=60 patients. The historical control group did not collect data on family carers, as they were not engaged as part of usual care, and therefore not available for recruitment. Therefore, the sample size for family carers will be n=30.

### Blinding and Randomization

Randomization is not possible due to the study design. The use of a historical control group for the pilot study was chosen to limit intervention contamination within the small rehabilitation unit as resources did not allow for 2 prospective cluster sites to be recruited. Blinding of participants and personnel to the intervention is not possible due to the nature of the intervention (nutrition counseling), study design of the historical control group (researchers not blinded in the historical control group), and lack of resources to fund blinded outcome assessments for the pilot intervention study.

### The FREER Intervention

By integrating formal and family care for malnourished rehabilitation patients, the FREER intervention aims to establish family carers as partners in the nutrition care team, thereby empowering and enabling them to manage and improve the efficacy of their preexisting nutrition-related care in the long term. In order to truly empower the family carer, the level of engagement between the dietitian, family carer, and patient in the FREER intervention model of care is derived from the Patterson, Kirk, and Wallace model, in which all team members have equal involvement and influence [21].

We have applied the four-step systematic approach for using the theoretical domains framework [22] to develop and establish the preliminary feasibility of the FREER intervention strategies. This was done through literature reviews [15-17], a qualitative study of family carer support needs and preferences [13], and stakeholder engagement (n=20 health care staff, unpublished). This pilot study will now establish preliminary efficacy and feasibility of the FREER intervention. All FREER intervention components will be delivered by the study dietitian and will use 3 individualized and needs-based strategies described in Multimedia Appendix I [23,24].

### Psychological Model of Behavior Change

Patient and family carer engagement strategies will apply the theory of planned behavior and reasoned action to increase an individual’s ability to make recommended changes [25]. Therefore, all engagements with the study dietitian will include education and shared goal setting, problem-solving, and contingency planning [25]. This model of behavior change was selected by the research team as it was considered the most appropriate to create partnerships with the patient and family carer, to lead to empowerment rather than dependency.

### Quantitative Outcome Measures

The selected quantitative outcome measures have been validated and previously piloted in the target population [10,12], and are outlined in Table 1. Nutritional status as defined by the PG-SGA numerical score [26] is the primary outcome (increasing score indicates increasing severity of PEM with typical scores 0-30). The PG-SGA was chosen in preference to the Mini Nutritional Assessment [27] and other nutrition assessment tools as both its score and categorization have the strongest criterion validity in this population and it has shown sensitivity to change in 1 week [1]. Secondary outcomes for the patient include (1) additional measures of nutritional status (PG-SGA rating of A, B or C), (2) energy and protein intake (kJ and grams per day), (3) mid-arm circumference (MAC), (4) physical function by Modified Barthel Index (MBI) [28], (5) Functional Independence Measure (FIM) [29], (6) body weight (kg), (7) health-related quality of life using a generic preference-based instrument (AQoL-6D) [30], (8) rehabilitation length of stay, (9) patient nutrition service satisfaction as per purpose developed Nutrition Service Satisfaction Survey modified from the Patient Satisfaction Survey with Inpatient Clinical Nutrition Services (Multimedia Appendix 2) [31], (10) 3 month rehospitalization (yes or no; and length of stay), (11) aged care admission (yes or no; and level of care). Secondary outcomes of the family carer are carer burden (Zarit Burden Interview Score [32]) and carer nutrition service satisfaction (Carer Nutrition Service Satisfaction Survey modified from the Patient Satisfaction Survey with Inpatient Clinical Nutrition Services and shown in Multimedia Appendix II) [31]. All assessment tools and physical measures for the patient will be completed by the study dietitian during patient interview, excepting the service satisfaction questionnaire which will be completed by the patient. All assessment tools for the family carer will be self-completed unless via telephone interview, or the carer has limitations with reading or writing.

The primary and secondary outcomes will be measured at baseline (recruitment T1), rehabilitation discharge (T2), and 12-weeks postdischarge (T3), as described in Table 1. Outcomes will be assessed at the rehabilitation site (T1 and T2) and a home visit or medical records as relevant (T3). If the participant is not able to be assessed at discharge at the rehabilitation site, T2 outcomes will be informed via telephone interview and medical records wherever possible; however, the physical measures including a component of the PG-SGA and the MAC would not be performed in this instance.

**Table 1:** FREER pilot study primary and secondary outcomes, assessment methods, and timepoints.

Outcome	Baseline (T1)	Discharge (T2)	Postdischarge (T3)	Measure and source of data
Nutritional status	X	X^a^	X	PG-SGA^b^ score and category Patient and carer interview
Weight (kg)	X	X	X	Calibrated study scales or medical records Patient interview
Energy intake (kJ)	X	X	X	24hr dietary recall Patient and carer interview
Protein intake (g)	X	X	X	24hr dietary recall Patient and carer interview
Mid-arm circumference	X	X	X	Tape measure Patient interview
Physical function	X	X	X	Modified Barthel Index Patient and carer interview supported by allied health care team Medical records
Physical function	X			Functional Independence Measure Medical records
Health-related quality of life	X	X^a^	X	AqoL-6D^c^ Patient interview
Patient nutrition satisfaction			X	Patient Satisfaction Survey Self or carer-completed
Rehabilitation length of stay		X		Medical records
Rehospitalization and length of stay			X	Medical records
Residential aged care admission			X	Medical records Patient or carer report
Carer burden		X	X	Zarit Burden Interview Self-completed
Carer nutrition satisfaction			X	Carer Satisfaction Survey Self-completed
Patient and carer experiences and perceptions of value^d^			X^e^	Qualitative interview conducted via telephone

^a^If T1 and T2 occur 6 days or less apart this measure will not be repeated as it will have assumed not to have significantly changed within that short time period as per feasibility data [10].

^b^PG-SGA: Patient Generated Subjective Global Assessment.

^c^AQoL-6D: Assessment of Quality of Life-6D.

^d^A subgroup of participants will be invited to participate by consecutive sampling with a target sample size of n=10 carers and n=10 patients.

^e^The interviews will be conducted up to 2 weeks following T3 by an independent researcher.

### Qualitative Outcome Measures

To understand the carer and patient experience and perception of the value of the FREER intervention, the first 10 participant pairs (both patient and carer, total n=20) who consent to be interviewed will participate in 30 to 60 minute semistructured interviews. Participants will be invited to participate in the interviews up to 2 weeks post T3 (Table 1) via telephone. The first interview with the patient and carer, who will be interviewed together if possible, will be an open discussion focused on topics identified in the literature and will be used to develop the semistructured interview schedule for the remaining interviews. The interviews will be recorded and analyzed qualitatively, using thematic analysis of verbatim interview transcripts based on the principals of grounded theory. For independence, the interviews will not be performed by nor in the presence of the study dietitian who implemented the FREER pilot intervention, and the interviews will be conducted and reported according to the Qualitative Research Review Guidelines (RATS) [33].

### Process Evaluation

A quantitative process evaluation will be simultaneously implemented. The intervention fidelity, intervention adaptions, and attrition rate will be recorded through researcher logs and voice-recorded telehealth consultations. Resources used to implement the process evaluation are outlined in Multimedia Appendix II.

### Adverse Events

As the patients are recognized to have acute or chronic morbidity requiring an inpatient admission, as well as being diagnosed with PEM at baseline, medical events and continued PEM are likely to be frequent as reflecting this medical and nutritional status. The FREER nutrition intervention reflects the current usual and best dietetic practice where only the method of engagement with patients and family carers is modified. Although nutritional treatment for PEM is considered low-risk adverse events may occur.

Adverse events possibly or directly related to FREER intervention methods will be recorded. These may be related to (1) nutrition-related biochemistry, (2) bowel habits, (3) allergic reactions to recommended foods and beverages, or (4) hydration status but will only be considered adverse events if status worsens from baseline. These intervention-related adverse events will be considered serious if they lead to the transfer from rehabilitation to acute care, additional intervention by the rehabilitation physician, or mortality.

### Ethical Considerations and Withdrawals

This study was approved by the North Coast New South Wales Human Research Ethics Committee (Approval HREC/18/NCC/47) and Governance (528N), as well as the Bond University Human Research Ethics Committee (528N-HREC/18/NCC/47). Written informed consent will be required for patients and family carers prior to their participation. Withdrawal of the patient or the carer from the study will cease the FREER intervention being delivered to both members of the caring pairs. However, the nonwithdrawing patient will continue to be asked to participate in outcome assessments if the carer withdraws from the study.

### Statistical Analysis

Intention-to-treat analysis will be used to evaluate quantitative outcome measures. However, those discharged home and those discharged to aged care will be reported separately. If the intervention group sample size is substantially smaller than the historical control group, cases will be matched to create a 1:1 ratio of control versus intervention. Outcomes and participant characteristics will be summarized via descriptive statistics. Changes in the control group over time have been previously analyzed and published [10]; however, the intervention group will be analyzed for change over time in continuous variables using linear mixed models, and chi-square tests for changes over time in categorical variables.

To determine the difference in primary and secondary outcomes between the intervention and control group over time, both continuous and categorical outcome variables will be analyzed via a marginal model using generalized estimating equations, with study group allocation and time in months as main predictors, and adjusting for baseline outcome measures.

## Results

Recruitment for the FREER pilot study began July 4, 2018, and is ongoing. At the time of manuscript submission, 14 participant pairs have been identified as eligible, with nine pairs (n=18 participants in total) consenting (preliminary recruitment rate is 9/14, 64%). The reasons for nonparticipation were family carer not interested (3/5, 60%) and patient not interested (2/5, 40%). Three family carers have withdrawn from the study at T2 (discharge). The stated reasons for withdrawal were (1) family carer being overwhelmed with caring duties (1/9, 11%), (2) family carer being overwhelmed with caring duties in the context of worsening carer health (1/9, 11%), and (3) changes to the family caring structure (1/9, 11%). Three of 9 (33%) patients were withdrawn following T2 because of (1) death due to the complication of a preexisting condition (2/3; 67%), and (2) geographical relocation out of the study area (1/3; 33%). The preliminary carer attrition rate is 33% (3/9) and the patient attrition rate is 33% (3/9).

## Discussion

Although compared with usual care, supportive nutrition interventions to increase dietary protein and energy intake in malnourished patients across all settings decrease all-cause mortality (risk ratio .78, 95% CI: 0.66-0.92, N=12 trials, N=6683 participants), the evidence is biased by poor quality study designs with limited translation of effective models of care to the clinical setting [2]. The pragmatic design of the FREER intervention will support translation to practice. Although the current pilot study has limitations related to study design, including the use of a historical control group causing selection bias and a lack of blinding, it will provide sufficient preliminary feasibility and efficacy data to inform the development of a future adequately powered, and well-designed RCT. In addition to the need for high-quality RCTs which evaluate nutrition interventions for malnourished older adults, research engaging family carers as part of the medical and nutritional care team is in demand [34,35]. By using a relevant framework [22] this research is designed to inform health service policies and will provide the foundations of future interventions translated into other health care settings and rehabilitation units.

## Appendix

Multimedia Appendix 1 The FREER intervention strategies.

Multimedia Appendix 2 FREER intervention and research materials.

## Abbreviations

AQoL-6D: Assessment of Quality of Life-6D
FREER: Family in Rehabilitation: EmpowERing Carers for improved nutrition outcomes
MAC: mid-arm circumference
PEM: protein-energy malnutrition
PG-SGA: Patient Generated Subjective Global Assessment
RCT: randomized controlled trial
