# Supplementary material for: Family in Rehabilitation, Empowering Carers for Improved Malnutrition Outcomes: Protocol for the FREER Pilot Study
Source: JMIR Res Protoc. 2019 Apr 30;8(4):e12647. doi: 10.2196/12647 (PMC6658316; doi:10.2196/12647)
Supplement: Multimedia Appendix 1 [file resprot_v8i4e12647_app1.pdf]

Appendix I: The FREER intervention strategies

1. **Nutrition counselling during rehabilitation admission**: The family carer will be engaged as an essential member of the nutrition care team for the malnourished patient by the study dietitian. The carer will be involved with: patient assessment; one or more collaborative nutrition counselling and education sessions focussed on improving patient nutritional status, based on need; and discharge planning. Guided by the nutrition care process [34], during nutrition counselling the study dietitian will collaboratively develop a nutrition care plan which provides highly individualised nutrition support strategies used to treat PEM, which are usually targeted towards enabling the patient to consume appropriate high protein-high energy foods to meet requirements. Counselling will also involve recommended strategies and resources to help overcome the individual's nutrition-related barriers (e.g. low appetite, food aversions, fatigue, poor dentition). The nutrition care plan will utilise a food-first approach; however, oral nutritional supplements may be provided if patients are unable to consume sufficient protein and energy by food alone. The intervention will be delivered by standard menu changes and additions via the rehabilitation foodservice as well as foods/beverages brought in by family carers. Patients and family carers will also be linked to other existing rehabilitation services as needed, such as speech pathology for modified food or fluid textures, or occupational therapy for modified eating utensils.

   Ideally the family carer will attend the rehabilitation site to participate in collaborative nutrition counselling; whereby paper-based resources will be provided. However, if the family carer is not able to attend the facility at the required time, they will be engaged via telephone either during or immediately following the patient consultation, and the resources will be left at the patient bedside, emailed, or mailed to them by post, according to carer

preference. Referrals made to health staff during rehabilitation admission will be made via the existing electronic referral system, and referrals to services or professionals outside of the rehabilitation site will be made as per the professionals preference. All intervention, referrals, and contacts will be recorded in patients' electronic medical records by the study dietitian. Study and intervention materials utilised during the rehabilitation unit are listed in Appendix II, and are available via Online Supplementary Material.

2. **Telehealth follow-up**: The patient and family carer will be planned to receive a minimum of four 15-30min telephone consultations from the study dietitian focussed on patient nutrition support, delivered over a period of 12-weeks post-discharge from rehabilitation. Consultation frequency and duration will be as needed, but will aim to be at least every three weeks. These telehealth consultations will involve a) either both the patient and carer, or b) be provided separately if patient and carer cannot attend the call together (patient consulted first). Consultations will be individualised and follow a semi-structured plan, guided by a call transcript (Appendix II and online supplementary material). Consultation content will follow the nutrition care process [34], and will be centred around nutritional monitoring, detailed food and nutrition-related problem-solving and contingency planning, modification of the individualised nutrition care plan for the patient, and linking with further supportive services such as home-delivered meals, provision or modification of oral nutritional supplements, or referral to other allied health professionals.

   If patients are discharged to a respite facility or residential aged care, the discharge and post-discharge intervention will be modified accordingly. This will involve the development of a patient handover summary to the facility and physician, and post-discharge telephone consultations with the family carer to assist them in ensuring their care-recipient receives the required follow-up during their residency, which will include being seen by an Accredited

Practising Dietitian if the patient remains malnourished at discharge. If the patient is discharged to an acute care facility, a patient handover will also be provided to the hospital's nutrition and dietetics department where usual care will be received. The telehealth intervention will commence once the patient is discharged home from hospital or respite.

3. **Resources**: An individualised selection of resources will be provided to and discussed with the patient and family carers to support day-to-day nutrition support and caregiving. During the first inpatient consultation with the study dietitian, patients and family carers will receive 1) a Nutrition Education Materials Online (NEMO) [35] high-protein high-energy diet appropriate to the individual (e.g. general, diabetes, vegan, pictorial), which includes a weight monitoring calendar, and 2) Individually written meal plans and goals (Appendix II). Additional NEMO Resources (e.g. modified texture diets, information for constipation) will be selected to suit the patients' and family carers' needs either during rehabilitation or post-discharge, via email or posted mail. The 'meal plan & recommendations' form will be updated as needed. To engage the support of the rehabilitation clinical team, a copy of the written meal plans/goals provided to patients will be added to their bed-chart and added to the medical records.

<u>Oral nutritional supplements</u>

As a pragmatic trial, supplements will be recommended to participants reflecting routine clinical practice which is on a case-by-case basis informed by patient-centred professional judgement; and the type, flavour, nutritional content, and volume of supplements recommended will vary according to individual needs, preferences, and food service availability. If supplements are recommended during the rehabilitation admission this will be via standard procedures whereby supplements are ordered as part of the food service, and the supplements chosen will be subject to food service stock availability and participant preference. Upon discharge, if supplements are recommended for an individual to consume at home or during an aged care admission, a seven-

day supply will be provided by the health service, and the patient will be registered with a nutrition supplement company to be able to purchase discounted supplements at their own expense, unless the expense is covered by the Department of Veteran Affairs. It will be up to the discretion of the patient/family carer to order and purchase the discounted supplements. If they choose not to purchase discounted supplements for consumption after rehabilitation discharge, the nutrition support recommendations will be modified to include alternative methods to improve energy and protein intake. Once the study has concluded, if patients require ongoing nutrition support and ongoing supplements, they will be referred to the government-funded community Accredited Practising Dietitian to access dietetic outpatient services and continue discounted supplements.
