# Supplementary material for: Family in Rehabilitation, Empowering Carers for Improved Malnutrition Outcomes: Protocol for the FREER Pilot Study
Source: JMIR Res Protoc. 2019 Apr 30;8(4):e12647. doi: 10.2196/12647 (PMC6658316; doi:10.2196/12647)
Supplement: Multimedia Appendix 2 [file resprot_v8i4e12647_app2.pdf]

Appendix II: FREER Intervention and Study Materials

Contents[a]

FREER intervention materials	Page
• FREER Consult Template	• 2
• FREER Medical Record Entry Template	• 3
• FREER Patient Referral Template (for referrals external to the rehabilitation unit)	• 4
• FREER Meal Plan & My Goals Template	• 5-7
• FREER Pilot Study Call Transcripts (to be used as a guide only)	• 8-14
• NEMO Resources available from www.health.qld.gov.au/nutrition (35)	• Available online only
**FREER research materials**	
• FREER Screening Flowchart	• 15
• FREER Researcher Process: Steps for T1, T2, and T3	• 16-18
• FREER Patient Nutrition Satisfaction Survey	• 19
• FREER Carer Nutrition Satisfaction Survey	• 20
**FREER process evaluation materials**	
• FREER Researcher Log	• 20-22

a. The materials listed here can be found in the Online Supplementary Material. Other outcome assessment tools have not been shared due to copyright, and other research tools such as consent forms have not been shared to ensure the privacy of researchers and participants.

FREER Consult Template

FREER ID	DOB	Age	MRN	Date

Reason for referral

Date of admission

Est. d/c

Presenting complaint

PMHx

Social

Carer ID M/F Age

Relationship Living together

Anthro

Wt

MAMC

Biochem

Clinical

PG-SGA
Bowels
Nausea
Vomiting
Swallowing
Chewing
Appetite

Mobility
Meds

EER

EPR

EFR

Diet

BF

MT

L

AT

D

S

LIKES

DISLIKES

Fluids

Assessment

Plan

FREER Medical Record Entry Template

Standard of documentation

First entry

Patient and their family carer eligible and consented to participate in FREER Pilot Study [date and time]. Patient and carer have retained a copy of the FREER Pilot Study Patient Information Form and completed consent form. Copy of patient completed consent form is in the medical record.

Reason for referral

Date of admission

Presenting complaint including hx if relevant

PMHx

Social Hx

Anthropometry

Biochemistry

Clinical

Nutrition Impact symptoms

Bowels

Nausea, vomiting

Swallowing/ chewing

Appetite

Mobility

Fluids

Meds

Recent test

Nut Requirements

Diet

Assessment

Plan

FREER Patient Referral Template

Patient Referral

To:	[Allied Health Professional]	From:	[Study Dietitian]
Patient name:		Date sent:	
DOB, age, gender:		Patient contact details:	

To [Allied Health Professional]

Thank you for seeing [name of patient] for [reason]. I have been seeing this patient following their admission to [Facility] Rehabilitation Unit, as they were diagnosed with malnutrition. I have been providing nutrition support to [name of patient] as part of the FREER Pilot Study undertaken by [researcher team affiliations].

[Details about patient goals, intervention and response].

[Further details about reason for referral].

Thank you in advance,

[Study dietitian name]
[affiliation]
[address]
[phone number]; [email]

FREER Pilot study:

**Title & study description.**	The FREER Pilot Study**:** **F**amily in **R**ehabilitation: **E**mpow**ER**ing carers for improved nutrition outcomes
**Study sites**	
**Chief investigator**	
**Ethics**	This study has received ethical approval from [details of approval]

FREER Meal Plan & My Goals Template
Date:

Patient name:

My Meal Plan

Breakfast

Morning tea

Midday meal

Afternoon tea

Evening meal

Supper/dessert

Date:

Patient name:

My Goals

1.

◯ **I did it!** ◯ **Almost!** ◯ **I'll try again next week**

2.

◯ **I did it!** ◯ **Almost!** ◯ **I'll try again next week**

3.

◯ **I did it!** ◯ **Almost!** ◯ **I'll try again next week**

4.

◯ **I did it!** ◯ **Almost!** ◯ **I'll try again next week**

5.

◯ **I did it!** ◯ **Almost!** ◯ **I'll try again next week**

Date: Patient name:

My Health Goals

1.

2.

3.

My Health Plan

1.

◯ **I did it!** ◯ **Almost!** ◯ **I'll try again next week**

2.

◯ **I did it!** ◯ **Almost!** ◯ **I'll try again next week**

3.

◯ **I did it!** ◯ **Almost!** ◯ **I'll try again next week**

4.

◯ **I did it!** ◯ **Almost!** ◯ **I'll try again next week**

5.

◯ **I did it!** ◯ **Almost!** ◯ **I'll try again next week**

FREER Pilot Study – Call Transcripts

Call 1 – Carer recruitment (optional – used if carer not met on ward)

- **Discuss** Introduction to research assistant, clinical need of patient
- **Discuss** Overview of FREER and participant of patient
- **Discuss** Carer PICF items and eligibility / consent

Introduction to RA, clinical need of patient

"Good morning/afternoon/evening, it's *<u>COACH NAME</u>* *here, I am a dietitian researcher based at [STUDY SITES], is this [<u>CARER NAME</u>]?*

How are you today?

[CARER NAME] I am calling because staff at the rehabilitation unit here have referred [PATIENT NAME] to me, as they have been identified as requiring intensive nutrition support to help their recovery.

Overview of FREER and participation of patient

The rehabilitation unit here usually provides some standard care, which involves one or more one-on-one discussions with the rehabilitation dietitian. However, at the moment [study sites] are conducting a study called the FREER study, basically to see if a more intensive nutrition support for the patient improves their recovery during and after rehabilitation.

This study has been discussed with [PATIENT NAME], and they [HAVE AGREED TO PARTICIPATE/ARE CONSIDERING PARTICIPATING], and therefore they gave me your number and permission to call you as they have said that you are involved in helping them with their normal daily activities a few times a week or more. For that reason, we would also like to keep you updated and involved in the nutrition support and in the study.

As [PATIENT NAME] is [KEEN TO/CONSIDERING] participation, are you happy for me to tell you a bit more about the study, to see if it is something you are interested in participating in?

Great!"

Carer PICF items and eligibility

[Read/discuss the the PICF to the carer]

If agree to participate:

- Arrange method for getting the PICF to the carer and obtaining consent
- Arrange time and method for the first nutrition counselling session once consent is arranged

If do not agree to participate:

"that is completely understandable, and I can confirm that non-participation will not affect the usual care provided to you and [PATIENT NAME]". However, it does mean that the [PATIENT NAME] is not able to participate in the FREER Study, and will not receive the telehealth support after discharge.

The next steps from here, is that [PATIENT NAME] will continue on their high energy high protein diet code, and I will ask the usual rehabilitation dietitian to see [PATIENT NAME] and provide nutrition support."

Conclude call

"Thank you very much for your time today. [confirm next meeting/appointment if relevant]. If you need it, my number is: [PROVIDE TELEPHONE NUMBER]. Thank you again, and goodbye".

Call ≥2 – Carer Updates during Rehabilitation Admission (optional – used if carer not present at nutrition counseling session/s)

- **Discuss** Overview of patient assessment / progress
- **Discuss** Overview of intervention
- **Discuss** Carer engagement and support

Overview of patient assessment / progress

"Good morning/afternoon/evening, it's COACH NAME here, I am the dietitian researcher based at [STUDY SITES], and providing care to [PATIENT NAME]?

[INITIAL CARER UPDATE]

On [DATE/TIME] I was able to meet with [PATIENT NAME], to do a full assessment and come up with a plan to support them during their rehabilitation admission. They have given me permission to call you to discuss the assessment and the plan.

[Give an overview of the current patient nutrition status, dietary intake, and nutrition concerns/problems].

[FOLLOW-UP CARER UPDATE/S]

On [DATE/TIME] I was able to meet with [PATIENT NAME], to see how they are going and modify the plan if needed to support them during their rehabilitation admission. They have given me permission to call you to discuss our meeting.

[Give an overview of the current patient nutrition status, dietary intake, and nutrition concerns/problems and progress towards goals].

How does my assessment of [PATIENT NAME] align with your observations, experiences and concerns?

Is there anything important you think that I might have missed, about their food and drink intake, health or anything else relevant?"

Overview of intervention

"Based on that assessment, [PATIENT NAME] and I have [come up with/modified] some goals.

[Outline the goals/changes to goals]

"Although [PATIENT NAME] and I developed these together, I'd like to know if you also have any other health or nutrition related goals for [PATIENT NAME].

[Modify/add goals as necessary].

"We have also [developed/modified] the plan to implement here in rehabilitation."

[Outline the plan and/or changes]

Carer engagement and support

"I would love to get your perspective on this plan, if you think these items are suitable for [PATIENT NAME], or any other thoughts? Can you foresee any barriers that [PATIENT NAME] may experience in receiving these interventions?

I would also like to know if you had any other ideas to meet [GOALS].

Finally, we have found that when the family carer is involved in helping to implement these interventions, that the outcome is much better. Although [PATIENT NAME] is in rehabilitation currently, we hoped you may be able to support the intervention by:

[Outline ways carer can support as per individualized plan. Usually – encouragement/support, bringing in food, assisting with opening packages/eating, etc].

[Check understanding/self-efficacy/stage of change and provide motivation/assurance/problem-solving/contingency plans].

Conclude call

"Thank you very much for your time today. Just to confirm, [review interventions]. *I will also [email/mail/leave at rehab] you a copy of [PATIENT NAME] [resources given].*

From here, I will [review the patient at X time, meet/call you to discuss the discharge plan, meet/call you for the first post-discharge follow-up call]. [confirm next meeting/appointment if relevant].

If you need it, my number is: [PROVIDE TELEPHONE NUMBER]. Thank you again, and goodbye".

Call ≥3 – Carer for discharge plan (optional – used if carer not present at nutrition counseling session/s)

- **Discuss** Overview of patient progress
- **Discuss** Overview of discharge plan
- **Discuss** Carer engagement and support

Overview of patient progress

"Good morning/afternoon/evening, it's <u>COACH NAME</u> here, I am the dietitian researcher based at [STUDY SITES], and providing care to [<u>PATIENT NAME</u>]?

As [PATIENT NAME] is being discharged to [DISCHARGE LOCATION], we have come up with a discharge plan. This is based on an observation of [give summary of patient progress/monitoring].

How does my assessment of [PATIENT NAME] align with your observations, experiences and concerns?

Is there anything important you think that I might have missed, about their food and drink intake, health or anything else relevant?"

Overview of discharge plan

"Based on that assessment and their progress to date, [PATIENT NAME] and I have developed some discharge goals.

[Outline the goals]

"Although [PATIENT NAME] and I developed these together, I'd like to know if you also have any other health or nutrition related goals for [PATIENT NAME].

[Modify/add goals as necessary].

"To reach these goals, we have developed a plan. As your contribution to this plan is vital, it is a draft only, and I would love you input and suggestions on it"

[Outline the plan: food & beverages, supplements, behaviours, self-monitoring, referrals]

Carer engagement and support

"I would love to get your perspective on this plan, if you think these items are suitable for [PATIENT NAME], or any other thoughts? Can you foresee any barriers that [PATIENT NAME] or you may experience in implementing these interventions?

I would also like to know if you had any other ideas to meet [GOALS].

Finally, we have found that when the family carer is involved in helping to implement these interventions, that the outcome is much better. We hoped you may be able to support the intervention by:

[Outline ways carer can support as per individualized plan. Usually – encouragement/support, bringing in food, assisting with opening packages/eating, cooking, shopping, engaging other family and friends to help, etc].

[Check understanding/self-efficacy/stage of change and provide motivation/assurance/problem-solving/contingency plans].

Conclude call

"Thank you very much for your time today. Just to confirm, [discharge plan]. *I will also [email/mail] you a copy of [PATIENT NAME] [resources given].*

As [PATIENT NAME] is now to be discharged, we will begin the telehealth follow-up component of the FREER Study. The first telehealth follow-up is planned for [two weeks after discharge/sooner if needed]. Ideally, it would be great to speak to both you and [PATIENT NAME] together at the same time.

[Discuss plans/options for this: i.e. Carer & patient at same house and on speaker phone/three-way telephone call].

[Set time, date, and reminder option for the telehealth call].

If you need it, my number is: [PROVIDE TELEPHONE NUMBER]. Thank you again, and goodbye".

Call ≥4 – Telehealth Option 1 – Patient alone OR Patient & Carer together

- **Discuss** Assess patient progress
- **Discuss** Modify/confirm intervention

Assess patient progress

"Good morning/afternoon/evening, it's COACH NAME here, I am the dietitian researcher based at [STUDY SITES], and providing care to [PATIENT NAME]?

Is now a good time to have our follow-up chat about how [PATIENTS NAME/YOU] are going since rehabilitation discharge?

Great!

[Review nutrition status using the ABCD Method: A: Anthropometry (e.g. weight change), B: biochemistry (e.g. any significant results from GP etc), C: Clinical (e.g. nutrition impact symptoms, changes to medications, other health professionals seen, physical function, social/family circumstance), D: dietary intake (brief diet history and comparison with goals/tolerance of supplements).

Is there anything else you think that I might have missed or relevant / anything significant happening for each of you?"

Modify / confirm intervention

"Based on what we have discussed, [confirm existing goals/modify existing goals].

[IF NEED TO MODIFY PLAN]

"To reach these goals [or solve a particular problem mentioned], do either of you have any ideas about what we could do/change? [Go on to develop a patient and carer-centered plan where all intervention items are developed together (e.g. food/beverage/supplement/ behaviours/social/family/referrals]. Also set monitoring plan].

[Provide updated resources and email/mail them to participant & carer]

[IF NO NEED TO MODIFY PLAN]

[provide encouragement, support, reinforce existing plan].

Conclude call

[OTHER TELEHEALTH APPOINTMENTS PLANNED]

"Thank you very much for your time today. Just to confirm, [plan & email/mail] *you a copy of [resources].*

[Discuss plans/options for next telehealth meeting: i.e. Carer & patient at same house and on speaker phone/three-way telephone call].

[Set time, date, and reminder option for the telehealth call – must be 3-weeks or less].

If you need it, my number is: [PROVIDE TELEPHONE NUMBER]. Thank you again, and goodbye".

[LAST TELEHEALTH APPOINTMENT]

"Thank you very much for your time today. Just to confirm, [plan & email/mail] *you a copy of [resources].*

This is our last planned telehealth consultation as part of the FREER study. I would like to thank you again for your support for this study, which will help us to design better support programs for rehabilitation patients.

However, we have one remaining item to do, our follow-up assessment to measure response to the program. This assessment will be in person at the [PATIENT NAME] residence. . If at this follow-up appointment we think [PATIENT NAME] may need some more nutrition support, I can arrange that with the community dietitian.

[Set time, date, and reminder option for the follow-up home visit – must be around 12-weeks post-discharge].

It would be great if [CARER NAME] could also be there for the follow-up assessment, but if not, I would like to email/mail you some questionnaires, to evaluate your experiences as a carer. I will include a reply paid envelope, or you can scan and email these to me.

If you need it, my number is: [PROVIDE TELEPHONE NUMBER] and email [PROVIDE EMAIL]. Thank you again, and goodbye".

Call ≥4 – Telehealth Option 2 – Carer Update (if patient received telehealth alone)

- **Discuss** Overview of patient progress
- **Discuss** Overview of intervention
- **Discuss** Carer engagement/support

"Good morning/afternoon/evening, it's COACH NAME here, I am the dietitian researcher based at [STUDY SITES], and providing care to [PATIENT NAME]?

On [DATE/TIME] I was able to chat to [PATIENT NAME] on the phone, to see how they are going and modify the plan if needed to support them since their hospital discharge. They have given me permission to call you to discuss our meeting.

[Give an overview of the current patient nutrition status, dietary intake, and nutrition concerns/problems and progress towards goals].

How does my assessment of [PATIENT NAME] align with your observations, experiences and concerns?

Is there anything important you think that I might have missed, about their food and drink intake, health or anything else relevant?"

Overview of intervention

"Based on that assessment, [PATIENT NAME] and I have [come up with/modified] some goals.

[Outline the goals/changes to goals]

"Although [PATIENT NAME] and I developed these together, I'd like to know if you also have any other health or nutrition related goals for [PATIENT NAME].

[Modify/add goals as necessary].

[We have also modified the plan for post-discharge nutrition support] OR [as the current plan seems to be working well, we haven't made any changes]

[Outline/confirm the plan and/or changes]

Carer engagement and support

"I would love to get your perspective on this plan, if you think these items are suitable for [PATIENT NAME], or any other thoughts? Can you foresee any barriers that [PATIENT NAME] may experience in implementing these interventions?

I would also like to know if you had any other ideas to meet [GOALS].

Finally, we have found that when the family carer is involved in helping to implement these interventions, that the outcome is much better. We hoped you may be able to support the intervention by:

[Outline ways carer can support as per individualized plan. Usually – encouragement/support, shopping, cooking, bringing food, assisting with opening packages/eating, engaging other family/friends, etc].

[Check understanding/self-efficacy/stage of change and provide motivation/assurance/problem-solving/contingency plans].

Conclude call

[OTHER TELEHEALTH APPOINTMENTS PLANNED]

"Thank you very much for your time today. Just to confirm, [plan & email/mail] *you a copy of [resources].*

[Discuss plans/options for next telehealth meeting: i.e. Carer & patient at same house and on speaker phone/three-way telephone call].

[Set time, date, and reminder option for the telehealth call – must be 3-weeks or less].

If you need it, my number is: [PROVIDE TELEPHONE NUMBER]. Thank you again, and goodbye".

[LAST TELEHEALTH APPOINTMENT]

"Thank you very much for your time today. Just to confirm, [plan & email/mail] *you a copy of [resources].*

This is our last planned telehealth consultation as part of the FREER study. I would like to thank you again for your support for this study, which will help us to design better support programs for rehabilitation patients.

However, we have one remaining item to do, our follow-up assessment to measure response to the program. This assessment will be in person at the [PATIENT NAME] residence. If at this follow-up appointment we think [PATIENT NAME] may need some more nutrition support, I can arrange that with the community dietitian.

[Set time, date, and reminder option for the follow-up home visit – must be around 12-weeks post-discharge].

It would be great if you could also be there for the follow-up assessment, but if not, I would like to email/mail you some questionnaires, to evaluate your experiences as a carer. I will include a reply paid envelope, or you can scan and email these to me.

If you need it, my number is: [PROVIDE TELEPHONE NUMBER] and email [PROVIDE EMAIL]. Thank you again, and goodbye".

FREER Pilot Study – Screening & Referral Flow Chart

1. According to medical notes and/or a brief chat, is the patient potentially eligible?

Inclusion criteria	Exclusion criteria
✓ At least 65 years of age.<br>✓ Likely to be malnourished based on:<br>o MST score ≥2<br>o ≥5% or 2-3kg weight loss<br>o Handover notes/ comments by staff/medical notes suggest it is likely<br>✓ Have a family carer:<br>o Medical notes indicate has a formal “carer”<br>o Medical notes/patient indicate that a family member or friend is providing support with any type of daily living<br>✓ Lived at home prior to admission.	✘ Does not live in the local area (e.g. on holidays/transferred from outside of NNSW district)<br>✘ Family carer is non-English speaking<br>✘ Likely to be discharged within 6-days<br>✘ Enteral or parenteral tube feed in place

2. Eligible?

Yes, I think so	No
✓ Refer patient to study dietitian.<br>✓ Place patient on HPHE diet code.<br>✓ Indicate the referral in the medical records.	✓ See patient as per usual

3. Confirmation

- The study dietitian will see the patient within 7 days and confirm eligibility. If eligible and consenting, the study dietitian will provide full care including monitoring and follow-up.
- If not eligible:
  - The patient does **not** require dietetic input – no further action taken.
  - The patient **does** require further dietetic input but is not eligible for the study or did not consent to the study – the patient will be referred back to the rehabilitation dietitian.

Referring to the study dietitian:

1. Write an email to [Study dietitian], APD: **[email address]**
2. OR make a note in the “FREER Study Referral List” held in the [Study Site] reception area.

FREER Researcher Process

FREER Researcher Process

STEPS	Timepoint 1		PAPERWORK
1.	Patient is referred via nursing staff, doctor or dietitian	OR patient is identified as potentially eligible from patient list on ward	- Recruitment log
2.	Electronic medical record (eMR) is reviewed for:<br>1. Age of patient<br>2. Place of residence (within 1.5hrs drive from rehabilitation unit)<br>3. Potential carer<br>4. Admission source (aged care facility/acute facility/home)<br>5. Discharge plan (in case conference notes)<br>6. Weight history<br>7. Signs of malnutrition (in observations and progress notes)		- Consult template
3.	If potentially eligible, continue to collect information required for initial assessment in eMR:<br>- PMHx<br>- Social<br>- MMSE/AMTS<br>- Relevant biochemistry<br>- Bowels<br>- Diet texture<br>- Mobility<br>- Medications	If not eligible, discuss with referral source and handover to local dietitian	- Consult template
4.	Conduct initial consult with patient and conduct PG-SGA		- Consult template<br>- PG-SGA
5.	If malnourished, explain study and continue to collect:<br>- 24-hr diet recall<br>- MAMC<br>- Physical function survey<br>- AQoL<br>- Education, pension, ethnicity, marital status, dentures<br>Explain PICF and leave a copy for patient and copy for caregiver to sign	If not malnourished, continue to complete dietetic assessment, appropriate intervention and progress note and handover to local dietitian	- 24hr recall<br>- Physical function survey<br>- AQoL<br>- Patient consent form<br>- Caregiver consent form<br>- Patient data collection form
6.	Collect FIM in folder on ward		- Physical function survey
7.	Complete progress note for participant, saved on eMR and USB<br>Change dietcode in eMR<br>Change midmeals/supplements in CBORD<br>Collect missing information from eMR (e.g. address, phone numbers)		- Researcher log for time with patient
8.	Call carer, explain study, organize a time to review with patient OR explain signature is need on consent form and will be collected – then phone call to caregiver after patient review	If caregiver is NOT willing/able to participate, then handover patient to local dietitian and withdraw patient from study.	- Researcher log for time with caregiver

STEPS	Timepoint 2		PAPERWORK
1.	Plan to review patient as close to discharge as possible, ideally with the carer present.	If patient has already discharged, contact patient to plan a time for a telephone appointment to complete T2	- Recruitment log<br>- Researcher log<br>- Discharge outcomes sheet
2.	Review eMR for:<br>1. Discharge plan (day, discharge location)<br>2. Discharge weight<br>3. Relevant biochemistry<br>4. Progress of nutrition impact symptoms<br>5. Dietary intake (if documented)		- Consult template
3.	See patient by bedside or in private consult room, as appropriate for the client.		- Consult template
4.	Using the nutrition care process, complete a full review of the patient.<br>Facilitate completion of questionnaires:<br>- PG-SGA<br>- 24hr diet recall<br>- Physical function survey<br>- aQoL	NB: If T2 is conducted via telephone, the physical examination in PG-SGA will not be applicable.<br><br>Ensure the patient has access to body weight scales at home and encourage to monitor weight as appropriate.	- Consult template<br>- PG-SGA<br>- 24hr recall<br>- Physical function survey<br>- AQoL<br>- Patient data collection form
5.	If patient does not yet have high protein high energy diet information, provide and discuss, ready for home. Together, develop goals suitable for the patient and carer.		- Relevant HPHE information sheet (Nutrition Education Materials Online)<br>- My goals sheet<br>- My Meal plan sheet
6.	Ensure the carer completes the Carer Burden Questionnaire. To reduce bias, the patient should not be present.		- Carer Burden Questionnaire
7.	If required, arrange with the patient (and carer) for home delivery of oral nutrition supplements. If so, create a patient profile and regime on the NCare website. Print the order slip and deliver to patient.<br>Arrange for a 1 week supply of nutrition supplements for the patient to take home on discharge, as per usual practice.	NB: if T2 is conducted by telephone, email the patient/carer the NCare order slip for them to call and arrange supplement delivery.	- Pink slip for take home supplements<br>- NCare patient profile and order slip with supplement regime
8.	Arrange time for T2.1 telephone call		- Recruitment log
9.	Complete eMR progress note for participant (save on FREER locked USB).		- Researcher log for time with patient
10.	If carer is not present at T2. Call carer, discuss progress, complete carer burden questionnaire, and arrange time for T2.1 phone call in 1-3 weeks as appropriate.		- Researcher log for time with carer

STEPS	Timepoint 3		PAPERWORK
1.	Visit the patient at their home (ideally with carer present). Bring a suitable snack to enjoy. Perform T3 on patient only if carer withdrew from study between T1 and T3.	If patient hospitalized, wait up to 2-weeks for patient to return home. If hospitalization >2-weeks, perform T3 at bedside in hospital.<br>If patient resides in aged care facility, perform T3 at aged care facility.	- Recruitment log<br>- Researcher log
2.	According to the Nutrition Care Process, complete a full dietetic review, including the following questionnaires:<br>- Patient information sheet (including weight, MAMC)<br>- PG-SGA<br>- 24hr diet recall<br>- Physical function survey<br>- aQoL<br>- Service satisfaction survey		- Consult template<br>- Patient information sheet<br>- PG-SGA<br>- 24hr diet recall<br>- Physical function survey<br>- aQoL<br>- Service satisfaction survey
3.	If required, refer patient to local dietetic services.		- List of community dietitians
4.	Ensure carer completes the Carer Burden Questionnaire.	If carer not present at T3, call the carer to discuss progress and complete Carer Burden Questionnaire.	- Carer Burden Questionnaire
5.	Thank the patient and carer for their participation. Invite patient and carer to participate in an evaluation interview with an external researcher.		

FREER Patient Nutrition Satisfaction Survey

Satisfaction with the Nutrition Services at the [Study Site]

Please tick the box that best reflects your response to the following statements:

Leave blank if not applicable.

	Strongly Agree	Agree	Neutral	Disagree	Strongly Disagree
I felt understood by the dietitian	❑	❑	❑	❑	❑
The care I received from the dietitian helped my body to heal	❑	❑	❑	❑	❑
The dietitian was polite & courteous	❑	❑	❑	❑	❑
The dietitian was attentive to my needs	❑	❑	❑	❑	❑
The care I received from the dietitian improved my general health	❑	❑	❑	❑	❑
The dietitian was well presented	❑	❑	❑	❑	❑
The dietitian listened carefully to what I had to say	❑	❑	❑	❑	❑
The care I received from the dietitian improved the results of my medical treatment	❑	❑	❑	❑	❑
The dietitian was friendly	❑	❑	❑	❑	❑
The dietitian came up with a good plan for helping me	❑	❑	❑	❑	❑
The care I received from the dietitian helped me to recover faster	❑	❑	❑	❑	❑
After I was discharged, I found the telephone calls from the dietitian were useful	❑	❑	❑	❑	❑
After I was discharged, I valued having continued support from the dietitian via telephone calls	❑	❑	❑	❑	❑
I was grateful that the dietitian kept my family / friend caregiver informed and involved	❑	❑	❑	❑	❑
	**Very poor**	**Poor**	**Okay**	**Good**	**Very good**
**Overall, how would you rate your satisfaction with the dietitian at the [Study site]?**	❑	❑	❑	❑	❑

Comments.

____

____

Date:

Participant number:

Thankyou

Modified for the FREER Pilot Study based on the Ferguson M et al (2001) Development of a patient satisfaction survey with inpatient clinical nutrition services. Aust J Nutr Diet **58**: 157-163.

FREER Carer Nutrition Satisfaction Survey

Satisfaction with the Nutrition Services at the [Study site]

Please tick the box that best reflects your response to the following statements:
Leave blank if not applicable.

	**Strongly Agree**	**Agree**	**Neutral**	**Disagree**	**Strongly Disagree**
I felt understood by the dietitian	❑	❑	❑	❑	❑
The care my care-recipient received from the dietitian helped their body to heal	❑	❑	❑	❑	❑
The dietitian was polite & courteous	❑	❑	❑	❑	❑
The dietitian was attentive to my needs	❑	❑	❑	❑	❑
The dietitian was attentive to my care-recipient's needs	❑	❑	❑	❑	❑
The care my care-recipient received from the dietitian improved their general health	❑	❑	❑	❑	❑
The dietitian was well presented	❑	❑	❑	❑	❑
The dietitian listened carefully to what I had to say	❑	❑	❑	❑	❑
The care my care-recipient received from the dietitian improved the results of their medical treatment	❑	❑	❑	❑	❑
The dietitian was friendly	❑	❑	❑	❑	❑
The dietitian came up with a good plan for helping me improve the quality of the care I provide to my care-recipient	❑	❑	❑	❑	❑
The care my care-recipient received from the dietitian helped them to recover faster	❑	❑	❑	❑	❑
After my care-recipient was discharged, I found the telephone calls from the dietitian were useful	❑	❑	❑	❑	❑
After my care-recipient was discharged, I valued having continued support from the dietitian via telephone calls	❑	❑	❑	❑	❑
I was grateful that the dietitian kept me informed and involved in my care-recipients nutrition support plan	❑	❑	❑	❑	❑
	**Very poor**	**Poor**	**Okay**	**Good**	**Very good**
**Overall, how would you rate your satisfaction with the dietitian at the [Study site]?**	❑	❑	❑	❑	❑

Comments.

____

____

Date:

Participant number:

Thankyou

Modified for the FREER Pilot Study based on the Ferguson M et al (2001) Development of a patient satisfaction survey with inpatient clinical nutrition services. Aust J Nutr Diet **58**: 157-163.

[illegible] participant number		[illegible] contact			
Carer participant number		Length of contact (mins)		Preparation time (mins)	
Persons present	• Patient<br>• Family Carer<br>• Others:<br><br>If patient only, carer followed up (date, method, length – also complete a new researcher log for carer contact):	Type of contact:	• Face-to-face at bedside<br>• Face-to-face in room at rehab<br>• Telephone call – during admission<br>• Telephone call – post discharge<br>• Call attempts before contact:<br>• Email<br>• Medical records review only		
Time-point	• Initial session<br>• Follow-up during admission (days post adm: _____________)<br>• Discharge session<br>• Post-discharge (days/weeks post adm: ______________)	Purpose of contact	• Perform assessment to inform intervention<br>• Provide nutrition intervention<br>• Provide nutrition education<br>• Provide support / encouragement<br>• Monitoring adherence/tolerance to intervention<br>• Monitoring response to intervention (anthro/bloods/symptoms/etc)<br>• Other, describe:		
Outcome of contact (inpatient only)	• Encouragement and support<br>• Setting/modification of goals<br>• Modification of food service diet code<br>• Modification of snacks/mid-meals<br>• Addition of ONS<br>• Modification of ONS<br>• Referral to other inpatient service: ________________<br>• Other:<br>• Resources provided (Describe):	ONS details (if relevant)	Kcal/ml:<br><br>Kcal/serve:<br><br>Protein/serve:<br><br>Serve size:<br><br>Flavour:<br><br>Brand:<br><br>Frequency:<br><br>If this is a modification, how so:		
Outcome of contact (after discharge	• Encouragement and support<br>• Problem-solving<br>• Modification of goals<br>• Modification foods/beverages	Comments, e.g. receptivity, health			

only)	recommended<br>• Modification of food/beverage frequency<br>• Modification of food/beverage serving size<br>• Self-monitoring plan<br>• Addition of ONS<br>• Modification of ONS<br>• Referral to other service: ______________________<br>• Other, describe:<br>• Resources provided (Describe what and how):	behaviour models used, problems solved, patient/carer concerns, other.	
Outcome: if admitted to RACF	• Care plan handed over<br>• Contact with staff, describe:<br>• Other:	Agreed next review	Day:<br>Time:<br>Method:<br>Items to review/discuss:
